# Supplementary material for: Causal Relationship between Aging and Anorexia Nervosa: A White-Matter-Microstructure-Mediated Mendelian Randomization Analysis
Source: Biomedicines. 2024 Aug 16;12(8):1874. doi: 10.3390/biomedicines12081874 (PMC11351342; doi:10.3390/biomedicines12081874)
Supplement: Supplementary file 1 [file biomedicines-12-01874-s001.zip › Supplementary Figure/Figure S8/Leaveoneout plot of MR analysis for TL to ubm-b-1471.pdf]

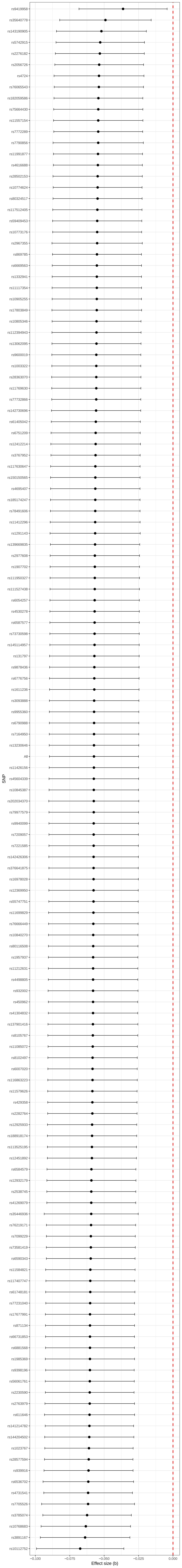
rs9419958
rs35640778
rs143190905
rs5742915
rs2276182
rs2056726
rs4724
rs76065543
rs182059586
rs75664430
rs11557154
rs7772289
rs7790856
rs11991877
rs4616688
rs28502153
rs10774624
rs80324517
rs117512405
rs59409453
rs10773176
rs2967355
rs869785
rs6669563
rs1332941
rs11117354
rs10005255
rs17803849
rs10805346
rs112394943
rs13062095
rs9600019
rs1003322
rs28363070
rs11769630
rs77732866
rs142730696
rs61405042
rs6751209
rs12412214
rs3767952
rs117630647
rs150150565
rs4695407
rs185174247
rs78491606
rs11412296
rs1291143
rs139669835
rs2977608
rs1907702
rs111950327
rs111527438
rs6054257
rs4530278
rs6587577
rs73730598
rs145114957
rs131797
rs9878436
rs6776756
rs1611236
rs3093888
rs9955360
rs6790988
rs7164950
rs13230646
All
rs11426156
rs45604339
rs10845387
rs202034370
rs79977579
rs9940099
rs7209057
rs7221585
rs142426306
rs376641875
rs16978028
rs12369950
rs55747751
rs11699829
rs76666449
rs10840270
rs80116508
rs1957937
rs11212631
rs4498805
rs932002
rs450962
rs41304832
rs137901416
rs8105767
rs11085072
rs8102497
rs6007020
rs116863223
rs11579626
rs429358
rs2282764
rs12925933
rs188918174
rs113525195
rs12451892
rs6584579
rs12932179
rs2538745
rs41269079
rs35446936
rs76219171
rs7099229
rs73581419
rs6590343
rs11584921
rs117407747
rs61748181
rs77231040
rs17677991
rs871134
rs66731853
rs6881568
rs1985369
rs9398196
rs56061761
rs2230590
rs2763979
rs611646
rs141214782
rs144204502
rs1023767
rs28577594
rs639916
rs6536702
rs4731541
rs7705526
rs3785074
rs10768683
rs3891167
rs10112752
SNP
-0.100
-0.075
-0.050
-0.025
0.000
Effect size (b)
